# Imaging presentations of foreign bodies that make for a challenging diagnosis: pictorial essay

**DOI:** 10.1590/0100-3984.2024.0057

**Published:** 2024-12-02

**Authors:** Daphne J. Theodorou, Stavroula J. Theodorou, Yousuke Kakitsubata

**Affiliations:** 1 Department of Radiology, General Hospital of Ioannina, Greece; 2 Department of Radiology, University Hospital of Ioannina, Greece; 3 Department of Radiology, Miyazaki Konan Hospital, Miyazaki, Japan

**Keywords:** Foreign bodies, Diagnostic imaging, Gastrointestinal tract, Respiratory system, Urogenital system., Corpos estranhos, Diagnóstico por imagem, Trato gastrointestinal, Sistema respiratório, Sistema urogenital.

## Abstract

Foreign bodies (FBs) can pose a diagnostic dilemma because a wide range of objects, comprising items incidentally detected or deliberately retained in the body, can be discovered on imaging investigations. Single or multiple FBs may be retained at different sites including the gastrointestinal tract, the genitourinary system, the respiratory tract, and the soft tissues, all of which warrant medical attention. More importantly, ensuing, serious complications related to harmful positioning of these objects can significantly hamper normal function of any involved organ system. Because various FBs may be detected throughout the body, it is important that radiologists are also familiar with a myriad of life-threatening complications associated with retained items, including impaction, obstruction, perforation, hemorrhage, embolization, chemical dissolution, poisoning, and sepsis. Imaging plays a key role in the detection, localization, and characterization of FBs. Radiologists need to describe in exhaustive detail suspected items with regard to the anatomical location, type, shape, and composition of the object under investigation. Clinicians can then predict whether the foreign object(s) will pass through the body uneventfully or need to be addressed in a surgical procedure.

## Abstract (pt)

Corpos estranhos (CEs) podem tornar-se um dilema diagnóstico, pois grande variedade de objetos, detectados incidentalmente ou deliberadamente retidos no corpo, pode ser descoberta em exames de imagem. Um ou vários CEs podem ser retidos em diferentes locais, incluindo o trato gastrointestinal, o sistema genitourinário, o sistema respiratório e os tecidos moles, e todos exigem atenção médica. Ainda mais importante é o fato de que complicações graves relacionadas ao sítio desses objetos podem prejudicar significativamente a função normal de qualquer sistema de órgãos envolvido. Mesmo que os diversos CEs possam ser detectados em todo o corpo, é importante que os radiologistas estejam familiarizados com as complicações com risco de vida, como impactação, obstrução, perfuração, hemorragia, embolização, dissolução química, envenenamento e sepse, associadas aos itens retidos. Os exames de imagem desempenham papel fundamental na detecção, localização e caracterização dos CEs. Os radiologistas precisam descrever em detalhes os itens suspeitos com relação à localização anatômica e ao tipo, forma e composição do objeto em investigação. Os médicos podem, então, prever se o objeto estranho passará pelo corpo sem intercorrências ou se precisará ser tratado por procedimento cirúrgico.

## INTRODUCTION

Foreign bodies (FBs) are objects that are alien to the body systems in which they are found, often jeopardizing organ integrity and function. A veritable galaxy of ingested and retained items can be responsible for hazardous or lethal complications. For example, complications of gastrointestinal (GI) tract FB ingestions account for approximately 1,500 deaths per year^**(1)**^. Specifically in children, ingestion of FBs is estimated to be responsible for 50-60 deaths per year^**(2)**^. Foreign objects are usually retained in the GI tract, respiratory tract, genitourinary (GU) system, or soft tissues. Given that asymptomatic cases account for up to 30% of clinical incidents involving an FB^**(3)**^, imaging is of fundamental importance in the detection of such objects. Conventional radiography continues to be the most appropriate initial test for the detection of FBs. Other imaging modalities, including fluoroscopy, sonography, computed tomography (CT), and magnetic resonance imaging have been helpful in evaluating FBs and the associated complications.

This article summarizes the imaging presentations of common and uncommon FBs at various anatomical locations. We illustrate a diverse array of FBs to familiarize radiologists with challenging clinical presentations.

## GI TRACT

A foreign object can enter the GI tract through the natural orifices via ingestion or deliberate insertion, as well as through iatrogenic misplacement. Although 80-90% of FBs will pass spontaneously, interventional removal of the retained foreign objects is required in 20% of the cases. Approximately 1% of patients need to undergo surgical removal of the lodged item^**(1)**^. A wide range of pathological conditions in the GI tract can be responsible for the retention of an FB, causing obstruction. Such conditions include peptic strictures; anastomoses or strictures secondary to chemical injury; sites of angulation or curvature; adhesions; gastric rings; webs; congenital deformities; motor disorders; and tumors. Failure of spontaneous, self-induced, or endoscopic dislodgement of an FB can cause serious complications, including abrasion, perforation, hemorrhage, fistulization, sepsis, or death^**(4-6)**^.

### Food bolus ingestion

**Figure 1:**
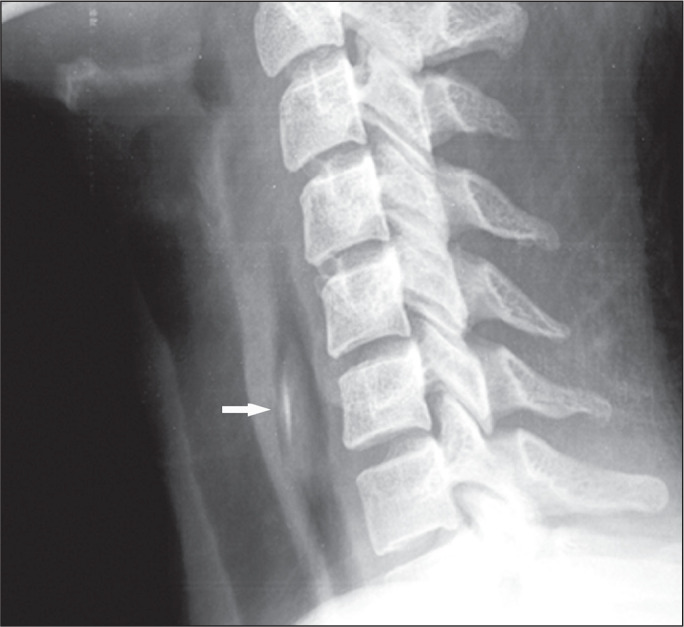
A 32-year-old woman who presented with coughing, choking, and sialorrhea after ingesting an appetizer containing a toothpick. Lateral radiograph of the neck, showing a large ingested food bolus and sharp wooden fragment (arrow) stuck in the cervical esophagus. Both items were removed endoscopically to prevent serious penetration injury.

**Figure 2:**
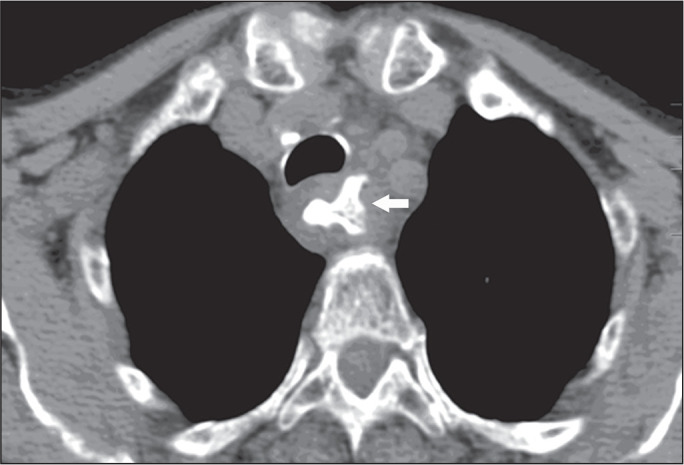
A 45-year-old woman who accidentally ingested a large lamb bone fragment. Axial CT image of the chest (mediastinal window) showing the impacted bone fragment (arrow) lodged in the dilated thoracic esophagus.

Frequently impacted alimentary FBs include red meat, poultry, bones, raw fruits, vegetables, fruit pits, and nuts. Edentulous patients and children commonly ingest poorly chewed food, seeds, and popcorn. Food bolus impaction may also occur in children who have previously undergone abdominal surgery or who have a congenital anomaly of the GI tract (e.g., pyloric stenosis and intestinal atresia). Chicken bones and fish bones typically lodge in the posterior hypopharynx and can be easily accessed by laryngoscopy. Alimentary bolus impaction typically occurs at the cricopharyngeus muscle, the narrowest point in the GI tract, measuring only 14 mm in diameter, which acts as the upper esophageal sphincter, and the cervical esophagus^**(7)**^. With swallowing, large foreign objects may exercise bolus pressure, passing through the cricopharyngeal sphincter. These forcefully ingested FBs may eventually pass the upper anatomic checkpoint and course to the esophagus and stomach (Figures 1 and 2). Even then, ingested objects with a diameter ≥ 2.5 cm or longer than 5 cm are rarely able to pass through the second anatomic checkpoint, at the pylorus^**(7)**^. Sequential sites of potential impaction include the duodenal sweep, the duodenojejunal flexure, and the ileocecal valve. Having reached the colon, an FB may lodge at the rectosigmoid junction, prohibiting its expulsion.

### Nonfood FB ingestion

**Figure 3:**
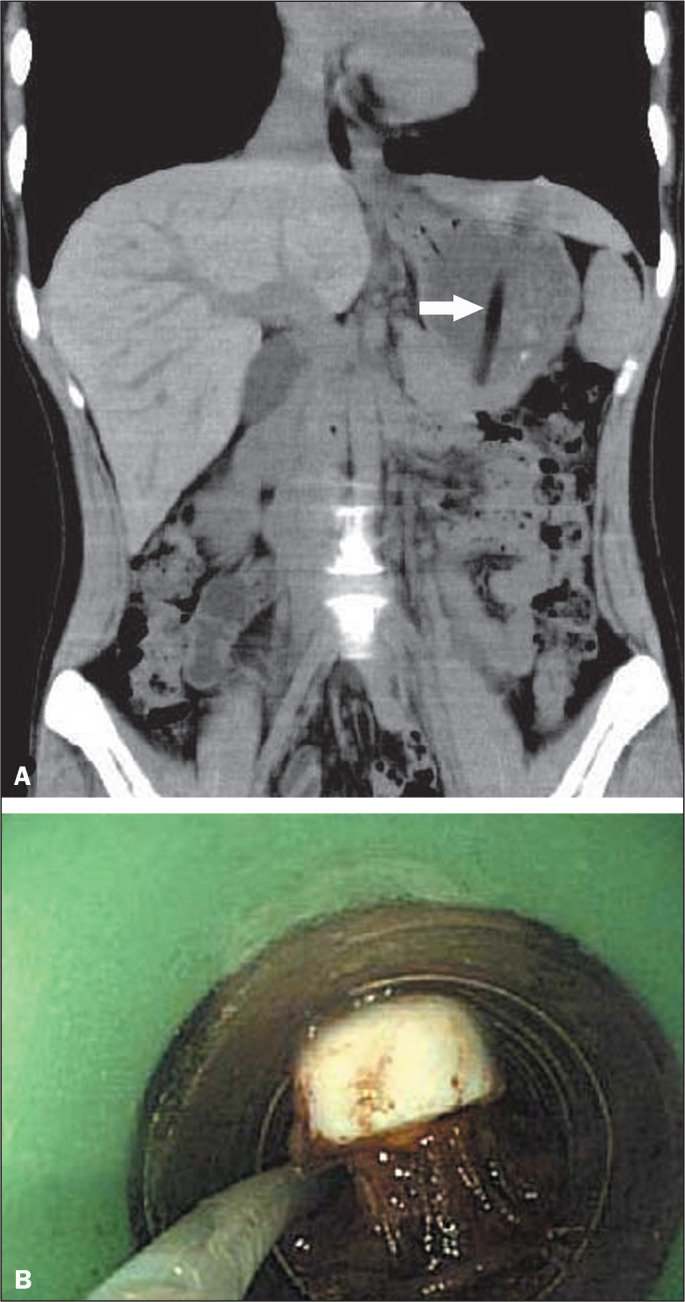
A 19-year-old woman who accidentally swallowed a toothbrush. A: Coronal reformatted CT image of the abdomen showing the toothbrush handle (arrow) lodged in the stomach. B: Photograph obtained during endoscopic removal of the item.

**Figure 4:**
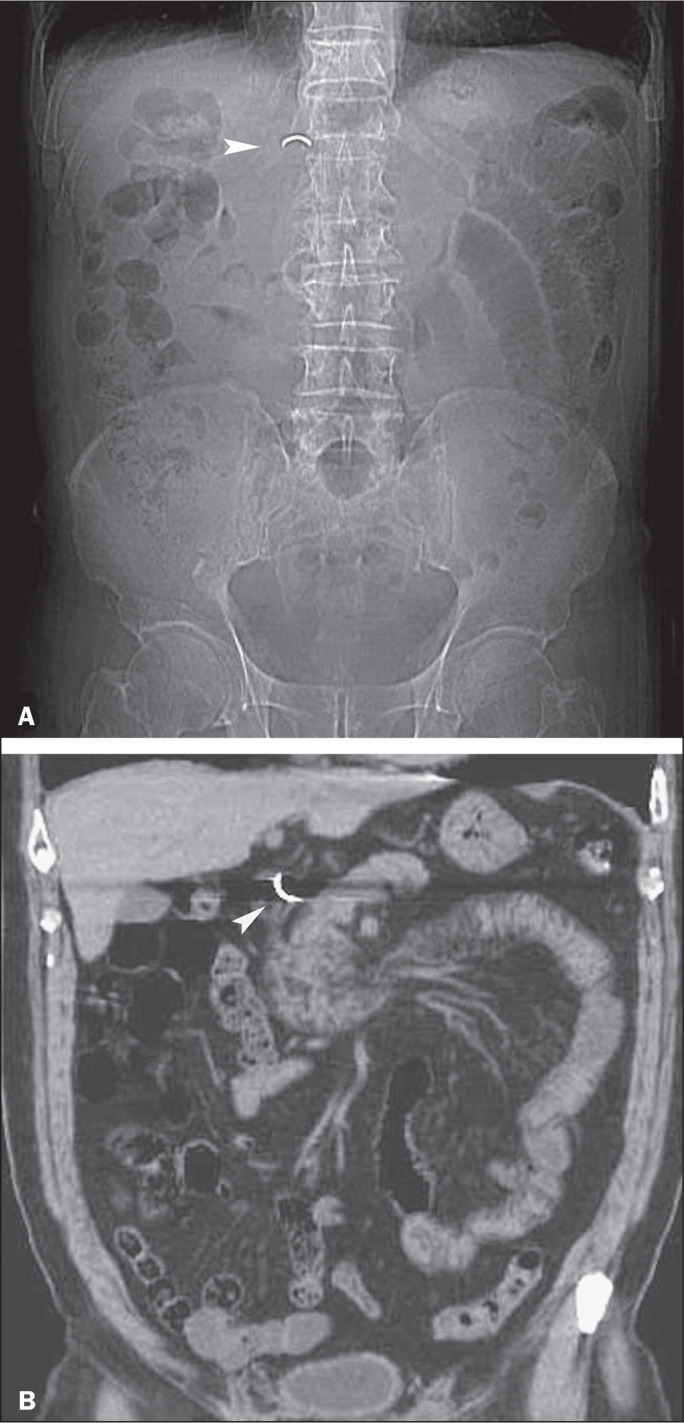
A 65-year-old woman who ingested a false tooth while eating peanuts. A: Anteroposterior radiograph of the abdomen showing a metal foreign object (arrowhead) most probably situated in the superior part of the duodenum. B: Coronal multiplanar reformatted CT image, obtained a few hours later, showing that the false tooth fragment (arrowhead) lodged near the superior duodenal flexure and having changed its orientation.

**Figure 5:**
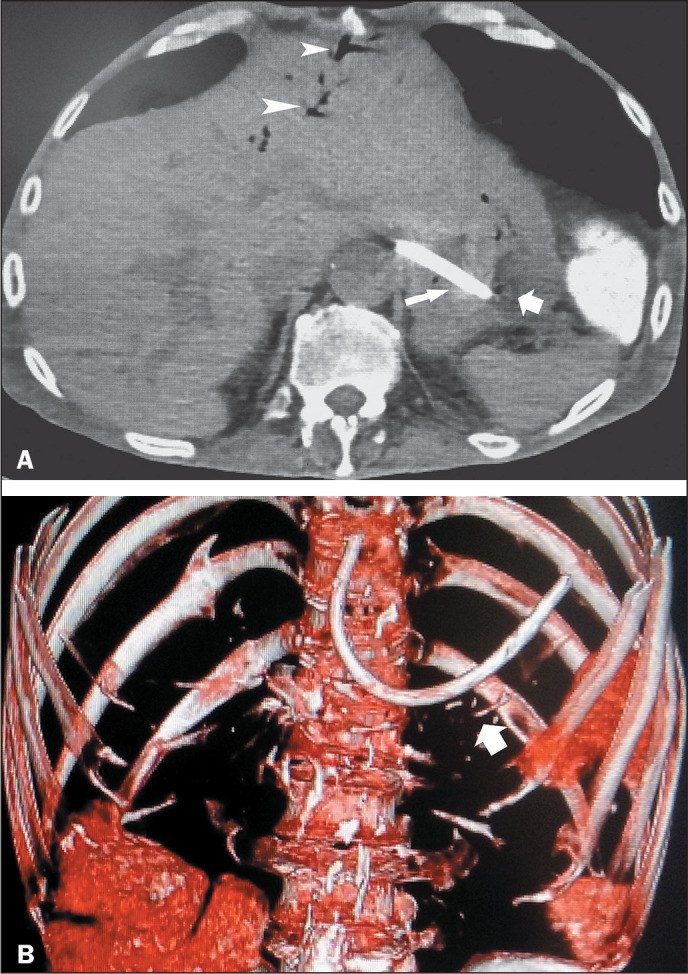
A 48-year-old male psychiatric patient who ingested a portion of an aerial television cable. A: Axial CT image showing the foreign object (thin arrow) that has penetrated the stomach wall. An air bubble is seen at the distal tip of the tubular item (thick arrow). Air in the intrahepatic bile ducts (arrowheads) in the left hepatic lobe implies penetration of the left bile duct during attempted swallowing of the cable. B: Coronal volume-rendering CT image (vessel view) showing the curvilinear cable (arrow) situated in the upper abdomen.

Pins, needles, small nails, brushes, wire bristles, razor blades, jewelry, beads, batteries, air gun pellets, fishing weights, lighters, plastic bags, paper clips, aluminum pop tabs, toothpicks, and marbles are among the most common hazardous, unexpected objects that can be accidentally or deliberately be ingested into the GI tract (Figure 3). Psychiatric patients, individuals with poor vision or inadequate dentition, and prisoners seeking secondary gains are at high risk for nonfood FB impaction, which can be detected with conventional radiography or CT (Figures 4 and 5). Other at-risk patient groups include individuals with poor mental status (e.g., those with dementia, alcohol dependence, or psychotropic substance use disorder), illicit drug users, and persons attempting suicide by swallowing caustic substances. Finally, iatrogenic causes of FB impaction in the esophagus may be associated with surgery, mediastinal radiotherapy, and prior chemical injury secondary to retained pills. Intellectually disabled patients and individuals with neuropsychiatric disorders may ingest multiple foreign objects, or consume non-edible items (e.g., metal objects, hair, stones, wood, and feces) in the frame of a serious eating disorder known as pica. If they reach the ileocecal valve, these deviant ingested FBs can obstruct the appendix, resulting in perforation or the formation of an appendiceal abscess^**(8)**^.

**Figure 6:**
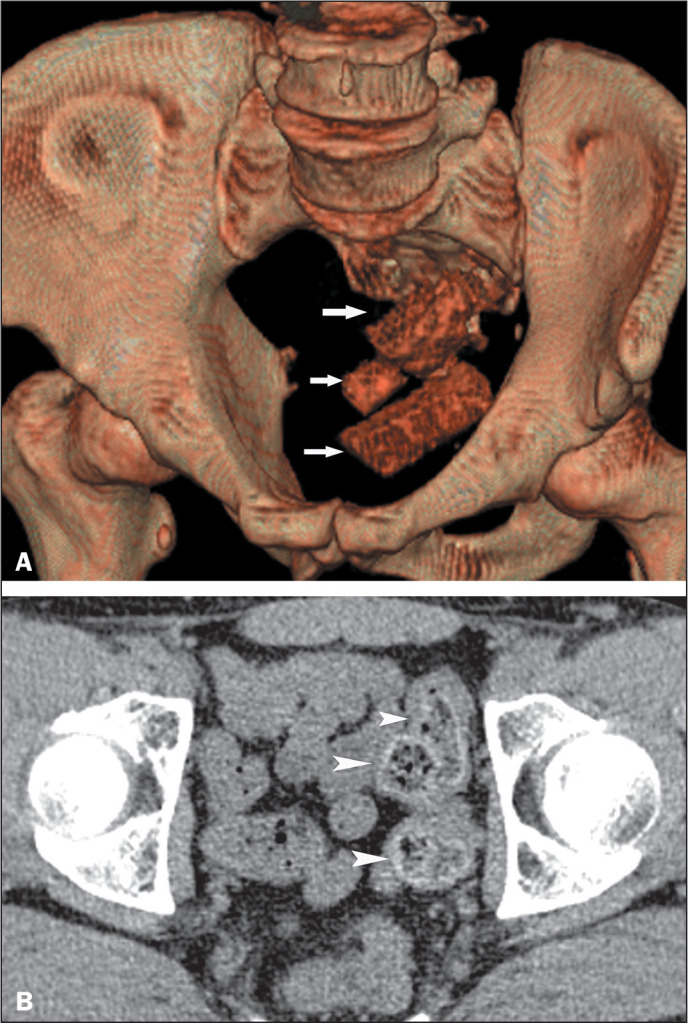
A 32-year-old male drug smuggler packing drugs to avoid arrest. A: Oblique volume-rendering CT image showing packets of drugs (arrows) purposefully deposited in the rectum. B: Axial CT image of the same drug smuggler, three years later, showing swallowed teabags stuffed with cocaine (arrowheads). Several drug packets were found in a stool analysis.

**Figure 7:**
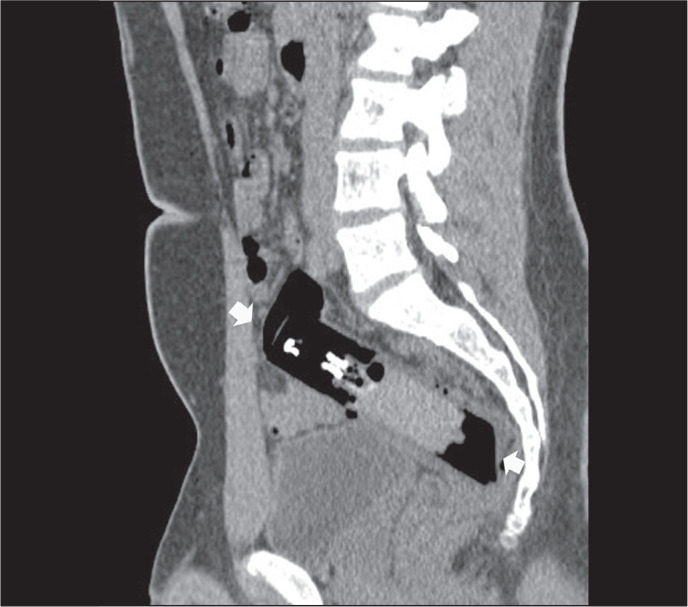
A 34-year-old male who inserted a sunscreen bottle in his anus during abusive sexual behavior. Sagittal reformatted CT image showing a large pump pressure spray bottle (arrows) impacted in the rectum.

In the lower GI tract, retained nonfood FBs can include objects introduced directly through the anus, with packets of illicit drugs typically being seen in drug smugglers who either ingest or insert them (Figure 6). Other FBs seen in the lower GI tract include rectal thermometers, plastic enema tubes, topical medication blisters, and suppository wrappers. The FBs inserted into the anus for autoeroticism reasons include vibrators, rubber or silicon sexual devices, fruit, vegetables, bottles, and jars (Figure 7). Although the detection of these unnatural items may be subject to humorous gossip, their retention and proximal migration can be associated with serious morbidity. Most importantly, the presence of rectal FBs in children is alarming because it raises the suspicion of sexual abuse. Following a procedure, iatrogenically introduced FBs such as surgical tools (e.g., clamps) and other items (e.g., gauze, sponges, and compresses, known as gossypibomas) can increase morbidity.

### FB ingestion in children

**Figure 8:**
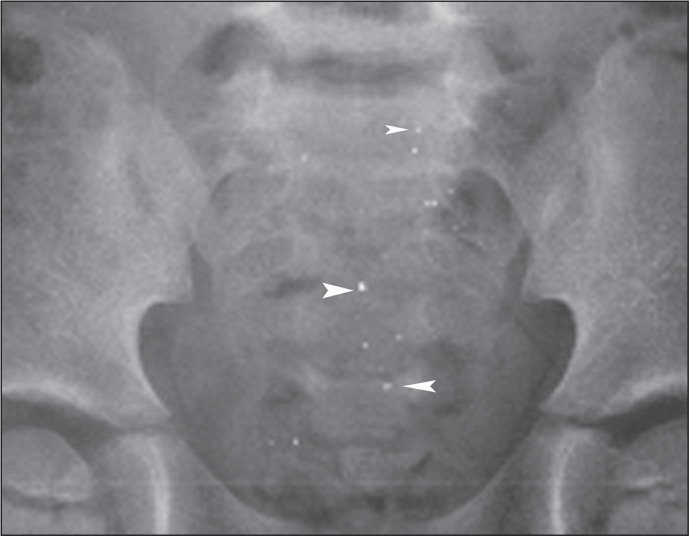
A 3-year-old female who swallowed metallic beads after breaking a mercury thermometer. Serial imaging was employed to visualize the passage of the mercury. Anteroposterior radiograph of the abdomen (magnified view) showing numerous tiny microbeads (arrowheads) moving with peristalsis and spreading throughout the distal small bowel.

**Figure 9:**
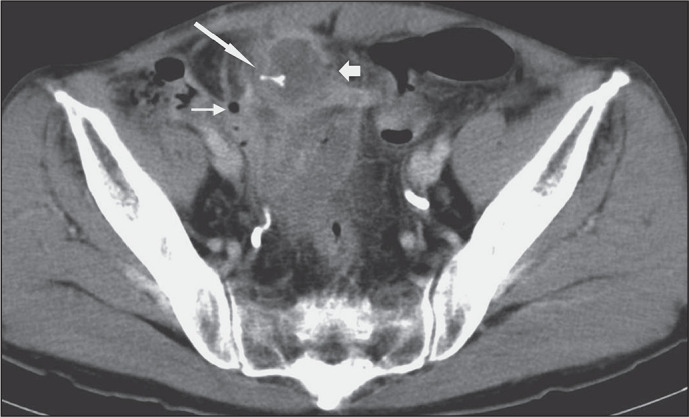
A 47-year old man with an abscess (thick arrow) in the small intestine due to ileal wall perforation by a chicken bone fragment (long arrow). Note the free gas (arrow) in an area of adjacent inflammation. The bony spike was removed by laparotomy.

**Figure 10:**
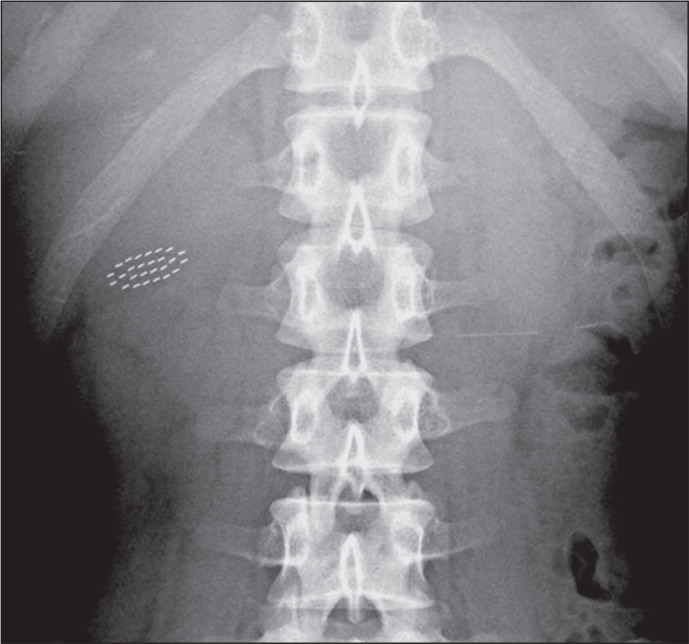
A 35-year-old intellectually disabled woman who swallowed a small toothbrush. On the anteroposterior radiograph of the abdomen, radiopaque bristles are seen in the right colic flexure. The brush passed without difficulty.

Annual reports have indicated that among 110,000 FB ingestions in the United States alone, 85% occurred in children, typically in those between 6 months and 5 years of age^**(9)**^. These urgent pediatric incidents involve various objects, with coins and button batteries usually being impacted in the proximal esophagus. A myriad of unforeseeable items may be ingested, including safety pins, button pins, buttons, plastic toys or toy parts, magnets, stones, and keys. Pediatric cases are alarming because ingested metal coins may contain corrosive chemical elements such as zinc or lead, whose prolonged retention can cause visceral abrasion and rupture, as well as systemic poisoning and death^**(9)**^. Similarly, ingested batteries can release heavy metals such as mercury and cadmium, as well as alkaline corrosive agents causing caustic injury, visceral ulceration, necrosis, and perforation at the site of entrapment^**(8)**^. As illustrated in Figure 8, mercury globules from a broken thermometer can be accidentally swallowed and can also cause poisoning^**(8)**^. Not infrequently, children ingest oral medications found in the bathroom medicine cabinet, as well as liquid or powder detergents^**(8)**^. Long, sharp items (e.g., needles, pins, fish bones, chicken bones, and toothpicks) can cause visceral penetration and may need to be surgically removed on an urgent basis (Figure 9). Finally, small size screws or nails, toothbrush bristles, press pins, and deciduous or permanent teeth can be naturally expelled, although their passing may need to be closely monitored on serial radiographs^**(10)**^, as depicted in Figure 10.

## RESPIRATORY TRACT

**Figure 11:**
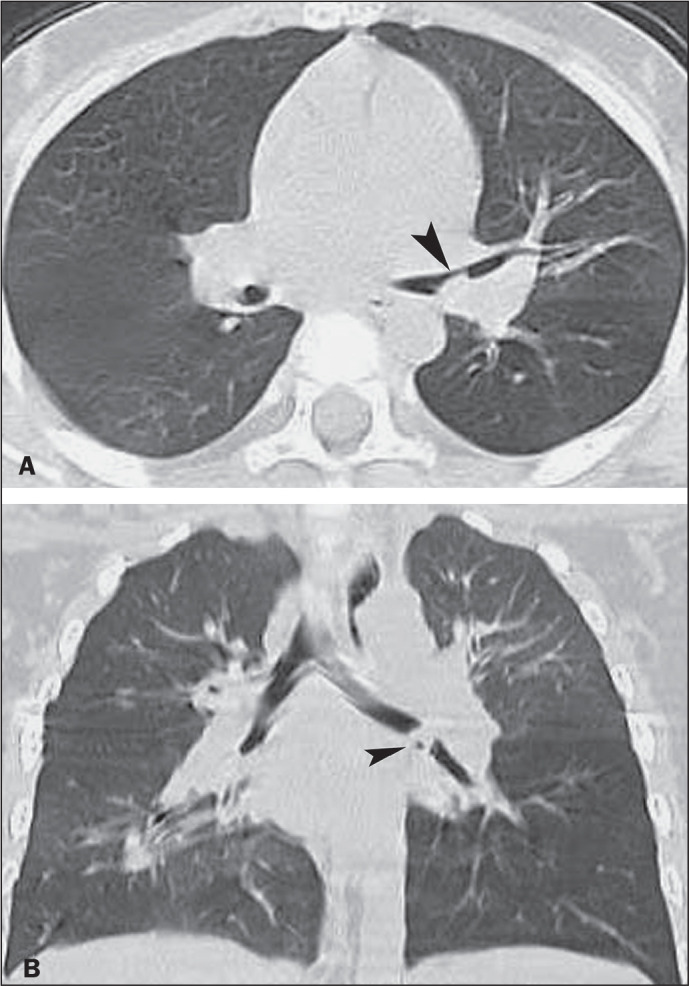
A 23-year-old man who presented with bronchospasm because of an aspirated roasted peanut. A: Axial CT image of the chest (lung window) showing partial obstruction of left main bronchus (arrowhead) due to the retained object. B: Coronal reformatted CT image showing near-total obstruction of the left main bronchus due to the impacted FB (arrowhead).

Inhaled, aspirated, or inserted FBs entering the respiratory tract may become lodged in the nose, throat, trachea, or bronchi. Obstruction of the tracheobronchial tree due to aspiration of peanuts, popcorn, food particles, fruit pits, plastic toy parts, teeth, stones, or sand is common in children, accounting for almost 3,500 deaths per year^**(9,10)**^. In adults, the aspirated foreign objects usually include peanuts, dentures, or tooth fillings, as shown on CT in Figure 11.

## GU SYSTEM

**Figure 12:**
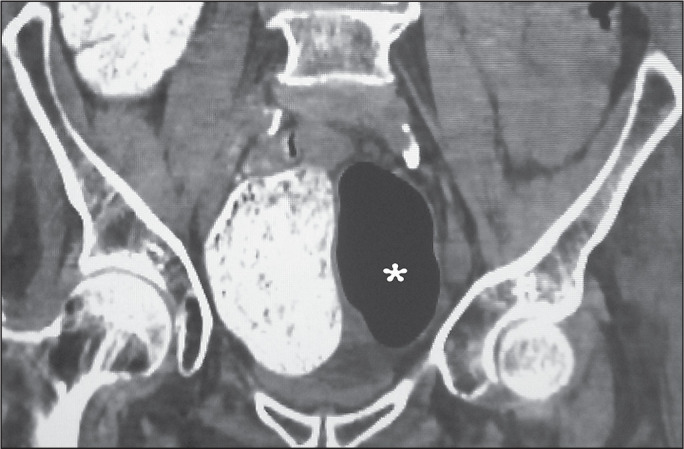
A 58-year-old man who engaged in sexually self-destructive behaviour by inserting an inflated balloon into the urinary bladder through the urethra. Coronal reformatted CT image of the pelvis shows the partially deflated balloon (asterisk). The large bowel is filled with an oral solution of gastrografin.

Similar to the rectal insertion of unexpected items, FBs detected in the vagina, urethra, or bladder are usually introduced on purpose by the patient (Figure 12). Other mechanisms associated with retention of FBs in the GU system include penetrating injury, surgery, and instrumentation. Items deposited in the GU system are more frequent in adults engaging in unusual sexual practices, mentally incapacitated individuals, and children^**(8)**^.

## SOFT TISSUE

**Figure 13:**
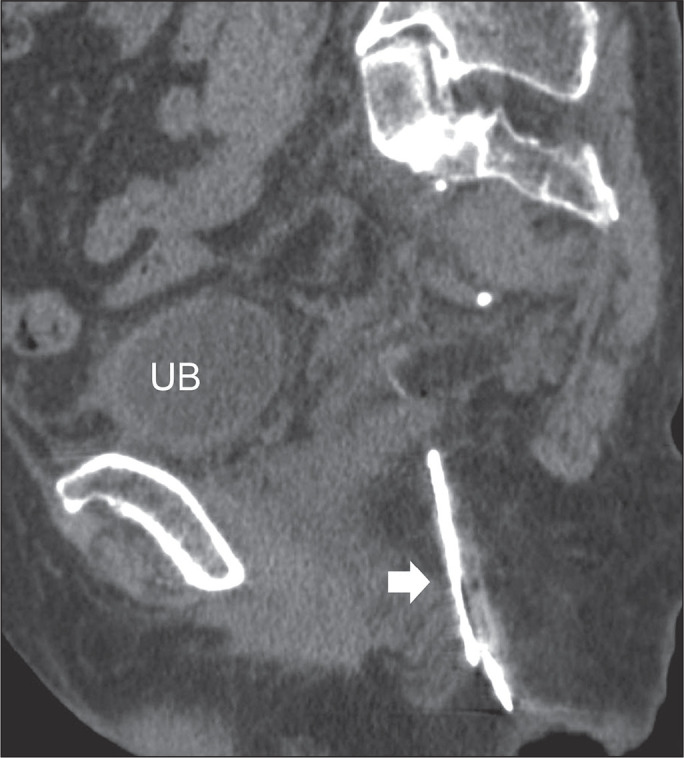
An 88-year-old woman with a bamboo splinter penetrating the right gluteal muscles after a fall. Sagittal reconstructed CT image delineating the full size of the FB (thick arrow) deeply impacted in the soft tissue. (UB, urinary bladder).

**Figure 14:**
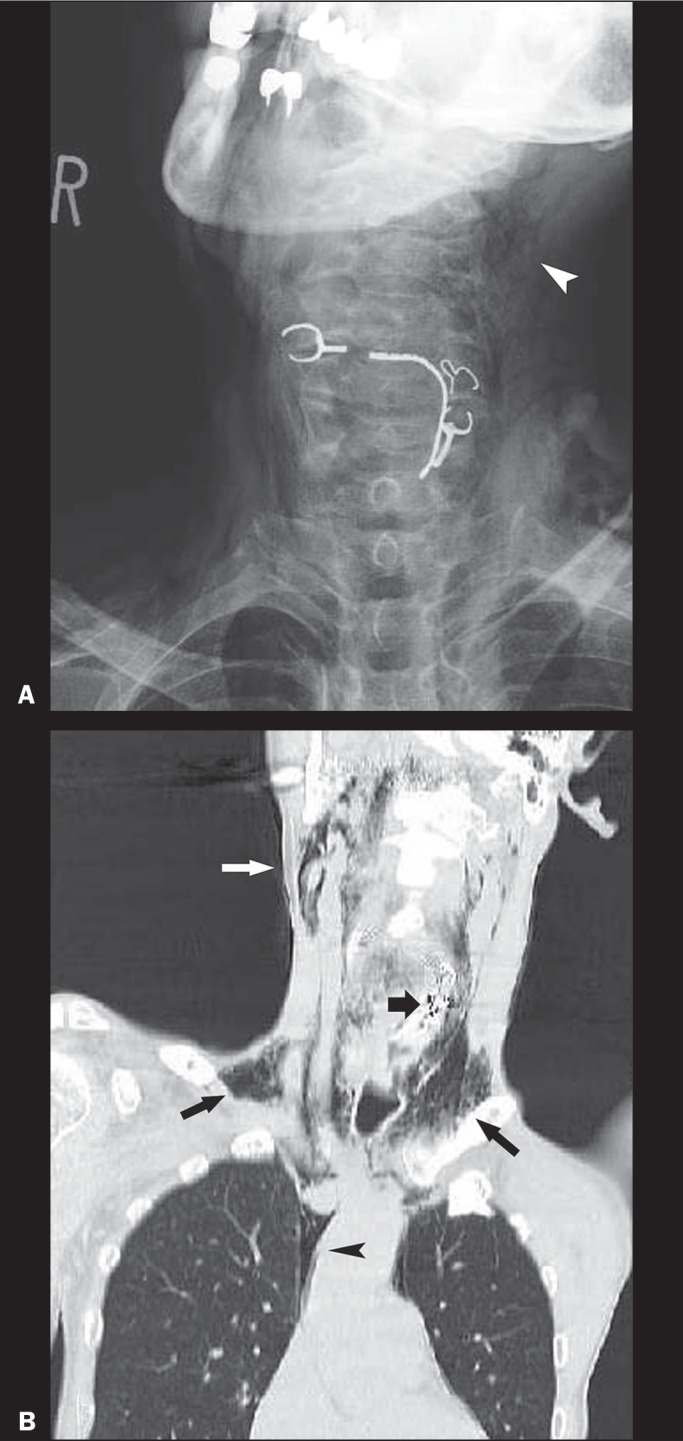
A 60-year-old man who presented with choking after having accidentally swallowed hooks and wires of a broken denture. A: Frontal radiograph of the neck showing the dislodged metallic dental prosthesis that has migrated from the mouth to the cervical esophagus, together with soft tissue emphysema indicating rupture of the esophagus (arrowhead). B: Coronal reformatted CT image (lung window) showing the metallic FB (thick arrow) in the neck, with soft tissue emphysema (white arrow) extending to the bilateral supraclavicular fossae (black arrows). Pneumomediastinum (arrowhead) is also seen.

Foreign objects are common in soft tissue, especially those affecting the superficial-most layers of the skin. As shown on CT in Figure 13, the FBs detected in soft tissue include plant material (e.g., wood, thorns, and splinters), various sharp-edged items, small pieces of glass, needles, nails, hammer drill metal bits, and bullets^**(7,8)**^. Incidents usually relate to traumatic injury, burns, and purposeful placement or accidental migration of items from the primary location of placement (Figure 14).

## CLINICAL PRESENTATION AND COMPLICATIONS

Symptoms and signs of FB ingestion in the GI tract are typically the result of obstruction. Esophageal impaction is far more common and may cause chest discomfort and dysphagia or odynophagia, choking, and persistent cough that prompt emergency treatment^**(7,8)**^. Fever, tachycardia, bloody saliva, and hematemesis are ominous signs that are indicative of perforation^**(7,10)**^. Colorectal retention of an FB can cause abdominal or rectal pain, bleeding, intussusception, and bowel obstruction^**(7,8,10)**^. Rectal perforation and peritonitis may complicate this scenario, because the affected individual could delay a visit to the hospital because of shame related to sexual or illegal drug-related activities^**(8-10)**^. Retention of an FB in the respiratory tract causing obstruction of the tracheobronchial tree can result in dyspnea, retrosternal pain, cough, and cyanosis^**(7,10)**^. In addition to asphyxiation, complications of FB aspiration include obstructive emphysema, pneumonia, mediastinitis, and fistula formation^**(9,10)**^. Retention of an FB in the GU tract may result in pelvic pain, bleeding, perforation, or infection^**(8,10)**^. Finally, the impaction of FBs in soft tissue can cause emphysema, superficial or deep-seated infection (phlegmon), or the formation of an abscess^**(7,8,10)**^.

## CONCLUSION

Throughout the body, retained FBs can be overlooked on clinical examination and imaging unless a high level of suspicion is maintained to make an early, accurate diagnosis. Thorough evaluation of diagnostic imaging examinations can allow the detection of various retained foreign objects. Detailed characterization of the associated serious complications can afford the best chance of a successful clinical outcome for patients.
